# Supplementary material for: Bolsa Familia Program and Perinatal Outcomes: NISAMI Cohort
Source: Int J Environ Res Public Health. 2022 Apr 28;19(9):5345. doi: 10.3390/ijerph19095345 (PMC9105772; doi:10.3390/ijerph19095345)
Supplement: Supplementary file 1 [file ijerph-19-05345-s001.zip › ijerph-1541600-supplementary.pdf]

Table S1 - Effect of the determinants of low birth weight according to different models. Santo Antônio de Jesus-BA. 2011-2018.

**Characteristics**	**Model 1**		**Model 2**		**Model 3**		**Model 4**		**Final Model**
	**OR (CI$_{95\%}$)**	**ORA (CI$_{95\%}$)**	**OR (CI$_{95\%}$)**	**ORA(CI$_{95\%}$)**	**OR (CI$_{95\%}$)**	**ORA(CI$_{95\%}$)**	**OR (CI$_{95\%}$)**	**ORA(CI$_{95\%}$)**	**ORA (CI$_{95\%}$)**
**Distal Level**									
Maternal Age									
18-24 years old	2.60 (1.27-5.29)	3.18 (1.47-6.90)		3.11 (1.43-6.75)		3.20 (1.48-6.90)		2.86 (1.17-7.00)	3.61 (1.53-8.53)
25-34 years old	1	1		1		1		1	1
≥ 35 years old	2.66 (0.89-7.89)	3.62 (1.17-11.19)		3.65 (1.17-11.35)		3.56 (1.15-10.97)		4.07 (1.14-14.47)	4.25 (1.20-15.06)
Family Income									
≥ 2 MS	1	1		1		1		1	1
≤ 2 MS	4.38 (1.54-12.41)	5.29 (1.81-15.40)		5.42 (1.84-15.97)		5.44 (1.87-15.83)		4.35 (1.44-13.09)	4.30 (1.45-12.71)
Skin Color									
Non-black women	1	1		1		1		1	
Black women	0.62 (0.30-1.30)	0.45 (0.21-0.97)		0.45 (0.21-0.97)		0.47 (0.22-1.00)		0.50 (0.20-1.24)	
Education Level									
≥ High school	1	1		1					
< High school	1.90 (0.79-4.58)	2.11 (0.80-5.57)		1.87 (0.67-5.22)					
Marital Status									
With partner	1	1							
Without partner	1.09 (0.50-2.41)	0.91 (0.40-2.08)							
Employment Status									
Active	1	1		1		1		1	1
Inactive	0.89 (4.88-1.63)	0.51 (0.26-1.00)		0.48 (0.24-0.94)		0.55 (0.28-1.07)		0.37 (0.17-0.83)	0.36 (0.17-0.78)
**Intermediate Level I**									
Smoking									
No			1	1					
Yes			1.52 (0.82-2.80)	1.26 (0.63-2.50)					
Alcohol comsuption									
No			1	1					
Yes			0.77 (0.40-1.49)	0.79 (0.38-1.61)					
**Intermediate Level II**									
Beneficiary of BFP									
Yes					1	1			
No					1.54 (0.46-5.09)	1.44 (0.42-4.91)			
**Proximal Level**									
Pregestational Anthropometric Status									
Low weight							1.93 (0.48-7.71)	1.76 (0.39-7.83)	
Appropriate							2.05 (0.87-4.83)	1.94 (0.75-5.02)	
Overweight							1	1	
Maternal Height									

Table S1 - Effect of the determinants of low birth weight according to different models. Santo Antônio de Jesus-BA. 2011-2018.

Characteristics	Model 1		Model 2		Model 3		Model 4		Final Model
	OR ($CI_{95\%}$)	ORA ($CI_{95\%}$)	OR ($CI_{95\%}$)	ORA($CI_{95\%}$)	OR ($CI_{95\%}$)	ORA($CI_{95\%}$)	OR ($CI_{95\%}$)	ORA($CI_{95\%}$)	ORA ($CI_{95\%}$)
≥ 1,50 m							1	1	1
< 1,50 m							2.74 (1.01-7.38)	3.22 (1.00-10.36)	4.08 (1-41-11.73)
Nutritional Orientation									
Yes							1	1	
No							1.03 (0.55-1.90)	0.93 (0.44-1.96)	

Table S2. - Effect of the determinants of prematurity according to different models. Santo Antônio de Jesus-BA. 2011-2018

Characteristics	Model 1		Model 2		Model 3		Model 4		Final Model
	OR($CI_{95\%}$)	ORA($CI_{95\%}$)	OR ($CI_{95\%}$)	ORA($CI_{95\%}$)	OR($CI_{95\%}$)	ORA($CI_{95\%}$)	OR ($CI_{95\%}$)	ORA ($CI_{95\%}$)	ORA($CI_{95\%}$)
**Distal Level**									
Maternal Age									
18-24 years old	1.89 (1.24-2.89)	1.71 (1.08-2.71)		1.89 (1.23-2.90)		1.89 (1.23-2.89)		2.02 (1.21-3.40)	1.88 (1.23-2.87)
25-34 years old	1	1		1		1		1	1
≥ 35 years old	1.03 (0.44-2.41)	1.09 (0.46-2.57)		1.10 (0.47-2.59)		1.03 (0.44-2.40)		1.33 (0.52-3.41)	1.03 (0.44-2.41)
Family Income									
≥ 2 MS	1	1							
≤ 2 MS	1.13 (0.72-1.79)	1.08 (0.67-1.75)							
Skin Color									
Non-black women	1	1		1		1		1	1
Black women	2.37 (1.17-4.80)	2.33 (1.10-4.95)		2.29 (1.13-4.65)		2.34 (1.15-4.75)		3.18 (1.25-8.06)	2.34 (1.15-4.74)
Education Level									
≥ High school	1	1							
< High school	1.17 (0.72-1.90)	0.97 (0.58-1.63)							
Marital Status									
With partner	1	1							
Without partner	1.38 (0,85-2.25)	1.03 (0.59-1.79)							
Employment Status									
Active	1	1							

Table S2. - Effect of the determinants of prematurity according to different models. Santo Antônio de Jesus-BA. 2011-2018

**Characteristics**	**Model 1**		**Model 2**		**Model 3**		**Model 4**		**Final Model**
	**OR($CI_{95\%}$)**	**ORA($CI_{95\%}$)**	**OR ($CI_{95\%}$)**	**ORA($CI_{95\%}$)**	**OR($CI_{95\%}$)**	**ORA($CI_{95\%}$)**	**OR ($CI_{95\%}$)**	**ORA ($CI_{95\%}$)**	**ORA($CI_{95\%}$)**
Inactive	1.17 (0.78-1.74)	1.02 (0.65-1.59)							
**Intermediate Level I**									
Smoking									
No			1	1					
Yes			0.75 (0.49-1.15)	0.78 (0.51-1.21)					
Alcohol comsuption									
No			1	1					
Yes			1.00 (0.60-1.45)	1.00 (0.59-1.43)					
**Intermediate Level II**									
Beneficiary of BFP									
Yes					1	1			
No					1.03 (0.53-2.00)	0.93 (0.48-1.83)			
**Proximal Level**									
Pregestational Anthropometric Status									
Low weight							0.87 (0.32-2.37)	0.71 (0.25-2.00)	
Appropriate							1.40 (0.85-2.30)	1.27 (0.74-2.16)	
Overweight							1	1	
Maternal Height									
≥ 1,50 m							1	1	
< 1,50 m							2.06 (0.99-4.26)	1.93 (0.85-4.39)	
Nutritional									
Orientation									
Yes							1	1	
No							1.40 (0.93-2.12)	1.38 (0.86-2.21)	
